# Template-Based Catalysis and the Emergence of Collectively Autocatalytic Systems

**DOI:** 10.3390/e28020184

**Published:** 2026-02-06

**Authors:** Roberto Serra, Marco Villani

**Affiliations:** 1 Department of Physics, Informatics and Mathematics, University of Modena and Reggio Emilia, 41121 Modena, Italy; rserra@unimore.it; 2 European Centre for Living Technology, 30123 Venice, Italy; 3 Institute of Advanced Studies, University of Amsterdam, 1012 WX Amsterdam, The Netherlands

**Keywords:** origin of life, Binary Polymer Model, protocells, dynamics, synchronization, sustainable replication

## Abstract

Mathematical and computational models, which have been successfully used in various fields of biology, are particularly relevant in studies on the origin of life, where wet experiments have not yet been able to obtain fully “living” entities from abiotic materials. This paper investigates mathematical and computational models of interacting polymers in prebiotic environments to understand how molecular replication and protocell reproduction could emerge. This study builds on the Binary Polymer Model (K-BPM), in which polymers are represented as binary strings that undergo catalyzed condensation and cleavage reactions, by introducing a biologically relevant variant (C-BPM), where catalytic activity depends on polymer structure. The model is analyzed with respect to the formation of autocatalytic networks, formalized as Reflexive Autocatalytic Food-generated (RAF) sets, embedded in a protocell in order to simulate their dynamics. The results show clear differences between K-BPM and C-BPM models. They also show that the existence of a RAF does not guarantee the survival of a population of protocells, although it may be possible when only a subset of the existing species partakes in the RAF, thus suggesting that small autocatalytic networks may have preceded the larger networks found in modern life.

## 1. Introduction

The vital processes of all known life forms largely rely upon the presence of long polymers, which are particularly abundant in living beings. We will therefore study, in this paper, dynamical models of the interaction between stylized polymers in prebiotic environments, paying particular attention to the conditions that allow molecular replication and reproduction of protocells (structures that are supposed to have existed at the dawn of life [1,2,3]).

Abstract dynamical models have been successfully used in biology to describe systems and processes at very different scales, from the molecular level to those of ecosystems and whole populations. Indeed, wet experiments have not yet been able to obtain fully “living” entities from abiotic materials, thus limiting the range of issues that may be experimentally explored, and making dynamical models particularly relevant in studies on the origin of life (OOL) [4,5,6,7,8], where great uncertainties still exist about how it happened on the Earth, and about how it may perhaps happen in a lab or elsewhere in the Universe.

Many interesting and intriguing hypotheses have been put forth, and quantitative models allow us to precisely define the hypotheses and to derive in-principle observable consequences that may be compared to field observations or to laboratory experiments. But models can do more than that; besides unfolding the consequences of specific hypotheses, they can also uncover “generic” properties [9,10], which are common to a wide set of otherwise competing proposals. A clear example is provided by the study of the emergent synchronization in protocells between the rate of duplication of the internal molecules and the rate of reproduction of the whole “container” [3,11]. Protocells are cell-like structures, much simpler than today’s cells, which should share with them at least (i) the presence of a semipermeable membrane which surrounds an internal part that hosts a number of chemicals (“replicators”) and their reactions and (ii) the possibility to reproduce, giving birth to new protocells. The synchronization between the rate of duplication of the internal molecules and the rate of reproduction of the container is a necessary condition to ensure sustainable growth of a population of protocells [3]. Such synchronization is guaranteed, in today’s cells, by sophisticated control mechanisms, which are unlikely to have been in place in prebiotic conditions.

By studying the behavior of dynamical models, it has indeed already been shown that synchronization does not require such control mechanisms, as it spontaneously appears under very different assumptions about the kinetic equations, which in turn implies that it can emerge under very different hypotheses about the chemical nature of the replicators and about their interaction laws. In previous works, it was shown that, generation after generation, protocells either spontaneously synchronize or their population dies out, a phenomenon which can be referred to as “starvation”. The growth of the protocell, which can eventually lead to its fission, takes place by synthesis of membrane lipids from precursors, which is catalyzed by the presence of internal chemicals. The interested reader may find a thorough discussion of these results in [12], where also the model of a growing and dividing protocell is described. The reaction–diffusion equations that are used here (as well as also in other works [3,12]) are briefly summarized in Appendix B.

The replication of some molecular species is at the heart of the key life processes of metabolism, whereby living beings continuously renovate themselves, and reproduction, where new extra copies of existing species are generated. In this paper we will model these processes, assuming that they are based on the action of polymers. Note also that the main biopolymers are “informational” ones, like polynucleotides and nucleic acids, where the presence of a particular type of building block at a certain position may (but not always does) determine the function that can be performed by that polymer. The chemical formula does not suffice to determine the properties of a polymer, since it is the sequence of building blocks (or at least a part of this sequence) that actually matters; for example, the sequence of nucleotides in RNA determines which amino acids will appear in the corresponding positions in the protein, while the sequence of amino acids in an enzyme can either determine its active site or contribute to define its spatial structure, which can also strongly influence its catalytic properties.

Enzymes, i.e., proteins endowed with catalytic properties, are informational polymers that directly affect the behavior of a cell. An intriguing model, able to describe how a population of possibly catalytic polymers changes in time [13,14,15,16,17], is sometimes called the Binary Polymer Model (BPM), since a polymer is represented by a binary string, i.e., a sequence of symbols taken from a binary alphabet. Polymers can be modified, either by condensation reactions (two strings are joined together) or by cleavages (a string is cut into two pieces). Reactions are assumed to require catalysts, so for example, a cleavage requires both a substrate and a catalyst, while a condensation requires two substrates and a catalyst. An interesting feature of this model is that a polymer can be both a substrate of a reaction and a catalyst of another reaction.

The BPM is obviously quite abstract and is therefore well-suited to deal only with some high-level questions, while it is unable to analyze specific issues related to the properties of the building blocks. For obvious reasons, binary polymers also cannot be used to explore the reasons (if any) that favor the choice of a four-letter alphabet for nucleic acids, and of a 20-letter one for proteins. On the other hand, as we shall see, the BPM can address questions related to the growth of the number of different species of polymers and to their survival in time, as new species appear or old ones get extinguished.

The classical BPM proposed by Kauffman (from now on, the K-BPM) will be described in Section 2. Since some slightly different versions exist, here we will concentrate for definiteness on the one described in detail in [3].

One aspect of the K-BPM that seems particularly questionable is that catalysts are associated with reactions fully at random. This is not a minor point, since the associations of catalysts with reactions determine the chemistry of the “world” of binary polymers that is described. Of course, in real systems it is the physic-chemical make-up of a molecule that determines which reactions it can catalyze, but in these abstract models—where the substrates are binary strings, and reactions are either condensations or cleavages—the possible worlds differ just by the coupling of catalysts to reactions. And here the K-BPM makes the radical assumption that, whenever a new type of polymer appears for the first time, it is associated at random with some reactions among those that are possible at that time. When new other molecular types are synthesized, the dice is thrown again, to decide which new reactions are catalyzed by the already existing polymer.

Enzymes, however, can catalyze some reactions and not others, depending upon their catalytic sites, their 3D shape and possibly other features, i.e., eventually upon their sequence, and similar enzymes can sometimes catalyze similar reactions [18], a feature that is completely lost in the K-BPM.

This limitation is not due to the high abstraction level of the BPM, but to the fact that any link between the structure of the molecule and its catalytic properties is completely lost; this may influence the way in which polymers can evolve, affecting the robustness of observed behaviors (in particular of autocatalytic sets, see below) with respect to mutations.

That’s why we also analyze here another model, which differs from the classical K-BPM in the way in which catalytic properties are attributed to molecular types. In this model, a polymer P can be defined as a possible catalyst, for cleavages or for condensations, and in this case a randomly chosen portion of its string is defined as its “active site”. If P is labeled as a catalyst for cleavage, it will catalyze cleavages of all the polymers that contain substrings that match its active site. In particular, complementary match is assumed here to be required, so for example, an active site like 100110 could cleave a sequence like 011001. For simplicity, we assume that the cleavage point is chosen, randomly, once and for all; for example, if this point is chosen between the second and third positions, 011001 will be cut separating 01 from 1001. These are just details; it might be possible to make different choices concerning the matching rule, or the position of the cut point (even choosing it at random), without severely affecting the main properties of the model.

If P is a catalyst for condensations, then it can join two strings whose endpoints match the active site (so 100110 can link a polymer ending with 011 to one starting with 001).

This modified BPM will hereafter be called C-BPM (complementary BPM), and it will also be presented in Section 2. Models based on a matching rule have been suggested by Bagley and Farmer [19], who, however, do not seem to have published the results of any dynamical simulation, and by Hordijk et al. [15,20], who have shown that particular autocatalytic systems (described in the following) can be present in these models. To the best of our knowledge, no wide analysis of the behavior of a binary model with active sites, comparable to the present one, has ever been published.

The two models presented here are based on different assumptions about the world they aim to simulate; these assumptions are reflected in the organization of chemical reactions systems they produce. We can name the set of chemicals, reactions, and catalysts as a “chemical reaction system”, or CRS for short.

The chemical worlds thus constructed, however, exist only potentially, in the sense that they are mere lists of all syntactically possible chemical species and reactions. However, even this first level of the model already allows for very useful observations.

Indeed, an important question addressed in this paper concerns the conditions that allow polymer replication (the other main question is related to the reproduction of protocells). This is summarized by the notion of autocatalytic cycles, which has a long history [3,9,10,21,22,23,24]. From a purely syntactic point of view (the simple presence of a closed, self-sustaining network of chemical species and reactions in a CRS), a useful tool for identifying an interesting set of chemical species and reactions is provided by the concept of a Reflexive Autocatalytic Food-generated set (RAF set, or RAF [15,25,26]), briefly summarized in Appendix A. RAFs are not necessarily cycles if the Food set (the set of chemical species whose presence is guaranteed by the environment) contains catalysts. But if this is not the case, as is usually assumed, then they must contain a cycle; it is then possible to associate RAF sets to the self-replication of molecular types and consider their existence as a necessary condition for the growth and replication of protocells. Interestingly, the probability of the presence of a RAF in a CRS and its structure are strongly influenced by the generation procedure of this chemical system (be it the K-BPM or C-BPM model—see Section 2).

The existence of a RAF in a CRS, however, is a necessary but not sufficient condition for observing a dynamically effective autocatalytic cycle; this is because some chemicals and reactions could disappear in the dynamical case.

It is therefore necessary to move to dynamical simulations and, to this aim, it is necessary to define a chemical–physical structure in which the reactions can actually take place. In this paper, we assume the presence of a protocell—a structure containing a membrane capable of retaining some of the chemical species inside while allowing others to pass through. Inside the aqueous interior of the protocell, chemical reactions (defined in the CRS) can occur, and chemical species can be produced and/or consumed, some of which can influence the growth of the membrane. Outside the protocell, we assume the presence (constant in this paper) of some chemical species capable of crossing the membrane. Of the entire CRS, only a few species are present at the beginning of the simulations; it is usually assumed that the internal species are chosen randomly from the set (or from a subset) of possible polymers and that the Food is composed of relatively simple polymers, for example, all those that do not exceed a certain length (the idea is that the environment does not continuously provide complex molecules).

It is reasonable to assume that the rate of a reaction depends on the concentrations of its reactants, for example, according to the well-known law of mass action. In this case, some catalysts might disappear from the system, for example, if they are also reactants in more frequent reactions. The system could then undergo complex changes, which could result in the absence of duplication of the protocell system, which we will explore in Section 3.

The study of these models was motivated by interest in OOL, so in Section 4, after studying the properties of the two types of models, we will discuss the implications of these properties for the study of prebiotic environments. Although a more in-depth discussion is deferred to Section 4, it is worth noting that RAF formation in K-BPMs strongly depends on a parameter *p*_r_ that determines the probability that a chosen polymer will catalyze an arbitrary reaction. In fact, comparing the model’s behavior for different *p*_r_ values, a phase transition can be observed in which the probability of a RAF’s presence suddenly shifts from nearly zero to nearly one—in this case, with the RAF’s size approaching the total number of possible species. In contrast, in C-BPMs, a more gradual increase is observed as the number of catalyzed reactions increases.

But determining the presence of RAFs does not suffice to determine the fate of the system. It has indeed been observed that the existence of a RAF does not imply that the corresponding dynamical models synchronize, since it can even lead to starvation. There are indications that relatively small RAFs readily lead to synchronization, while this may not be the case for large RAFs. This suggests that further studies could explore the conditions that could allow a gradual increase in RAF size, a possibility that will also be outlined in Section 4.

## 2. The Modified BPM

### 2.1. Introduction

In this paper, we aim to simulate protocellular systems, whose artificial chemistries can host catalysts, whose activity depends on the structure of the involved chemical species. To proceed, we therefore need to (i) characterize the artificial chemistry and (ii) decide how to organize the protocellular system (shape, presence of a membrane, list of chemical species capable of crossing the membrane, kinetic constants).

The construction of the laws of the “artificial chemistry” by the “classical” K-BPM model resorts to the random generation of catalytic reactions. In this paper, we aim to highlight the consequences on the dynamics of choosing another generative criterion, which involves the structure of the molecules involved in the reaction (Section 2.2).

The chemical world thus constructed exists only *in potential*, in the sense that it is a pure list of all possible chemical species and of their possible reactions. However, this part of the model already allows for very useful observations; in particular, we will see that the organization of the worlds generated by the K-BMP model is very different from that present in the worlds generated by the C-BPM model (Section 2.3). Furthermore, the latter can much more easily accommodate autocatalytic groups of substances and chemical reactions (RAF)—Section 2.4.

Finally, it is possible to observe the influence that the action of structure-dependent catalysis has on the chemical types involved in RAFs, particularly if RAFs are not large (Section 2.5).

### 2.2. The Construction Algorithms

In the BPM model, polymers are represented by ordered strings of length L, whose elements (monomers) are taken from a two-letter alphabet, for example {A, B}: there are therefore 2^L^ different types of strings (monomers and polymers) that are L monomers long. A condensation reaction joins two polymers, provided that the catalyst for this reaction exists. If we have polymers P_D_ and P_E_, we then obtain:

P_D_ + P_E_ + C → P_D_P_E_ + C

where C is the catalyst (whose number of molecules does not change).

A cleavage reaction cuts a P_1_P_2_ polymer into two shorter parts, provided that the catalyst for this reaction exists:

P_1_P_2_ + C → P_1_ + P_2_ + C

There are in principle two ways to generate artificial chemistries. One approach consists of considering all possible reactions among all possible chemical species and generating a random number between 0 and 1. If this number is less than the threshold probability *p_r_*, a catalyst is randomly chosen, and the reaction is effectively inserted into the current instance. The potentially infinite (or at least enormous) number of chemical species leads, however, to practical difficulties, as they require a lot of computational power and memory.

The second approach allows the exploration of these immense potential chemistries, without having to generate them in their entirety. The method starts from an initial set of chemical species (called a “firing disk”) of maximum length *FD_Lmax_* and calculates the number of reactions to be inserted (equal to the number of current species, multiplied by *p*), by randomly choosing a catalyst from the set of existing species, possibly including those that have been generated ex novo by the newly added reactions (remember that only catalyzed reactions are possible). This way, new chemical species are generated, whose length can even exceed *FD_Lmax_* monomers. The process iterates, recalculating the number of possible reactions, realizing the missing fraction *p_r_* of them, and thus creating other species, until no more new species are created. The important factors are the probability pr that a reaction is catalyzed and the number of initial species; combinations of these can lead to subcritical situations (in which nothing happens), supercritical situations (in which there is infinite expansion) or critical situations (the separating zone between the two previous cases, in which there is the possibility of having chemistries of non-negligible but finite dimensions) [14,27].

Finally, it is possible to introduce a constraint regarding the maximum length of the species that can be constructed. The molecular weight of a linear polymer is in fact an increasing function of its length, and quite often very long molecules precipitate and are no longer available for further reactions (which is usually assumed to take place in a fluid). All this translates into a limit on the maximum length *L_max_* of a molecule reactions that lead to larger molecules can be carried out (but the fate of the products is ignored) or more simply prohibited. In this case, the maximum number of legal polymers is therefore 2*^Lmax^*^+1^ − 2.

The initial composition of the firing disk can be chosen in several ways, for example, all monomers and all short polymers whose length does not exceed a certain threshold, or a similar set from which some species may be missing. The rationale is that the environment typically provides simple substances (in the representation used, short strings) that can be condensed to produce more complex species.

The classical BPM (K-BPM) and the modified BPM (C-BPM) differ in the association rule between catalysts and catalyzed reactions; in the former case, this occurs randomly, usually with uniform probability [13,14] or possibly with a length-dependent probability [15]. In the C-BPM model, each catalyst is assumed to host an “active site”, which intervenes in the selection of reactants. A model of this kind has been considered by [28].

**Figure 1:**
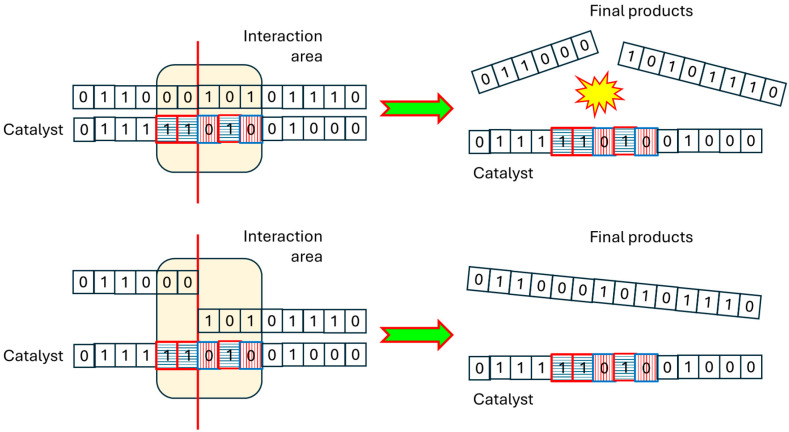
Template-based cleavage and condensation. (**Top**) cleavage. The catalyst contains an active site (shown as colored monomers, and highlighted by the ochre box) and a designated cleavage position (the vertical red line). A molecule is treated as a reactant when a segment within it is complementary to the catalyst’s active site; once this match is found, the molecule is split (the break is highlighted in the figure by a small “explosion”) at the cut position. (**Bottom**) condensation. The catalyst again features an active site and a suture position. A molecule qualifies as the first reactant if its ending segment complements the first portion of the active site, and another molecule serves as the second reactant if its starting segment complements the second portion of the active site.

To precisely define the C-BPM model, further details are needed; in this work, we assume that each newly generated species is as a catalyst (for cleavage or condensation) or not. If a species is a catalyst, a substring (the “active site”) of length *L_smin_ ≤* Λ *≤ L_smax_* is identified within the string representing it; within this substring, a position is randomly chosen where the cut (in the case of cleavage) or the suture (in the case of condensation) will occur. In the case of cleavage, all existing polymers will be scanned for complementary substrings. The definition of complementarity is obvious in the case of binary polymers (i.e., an A must correspond to a B, and vice versa), while it would need a more elaborate definition if more than two types of building blocks were allowed; in this paper, two (sub)strings are complementary if and only if all their monomers are complementary (note that in this approach, only polymers at least as long as the active site can be catalysts). If identified, the polymer will be cleaved, generating either existing species or new species. In the case of condensation, all ordered pairs of polymers will be considered, looking for those cases where the concatenation of the terminal part M_1_ of one molecule is complementary to the first part of the active site, and the initial part M_2_ of another is complementary to the second part of the active site. In this case, a condensation occurs, generating the species R-M_1_M_2_-R, with R indicating the remaining part of the involved species, possibly empty (see Figure 1). In general, it is possible that the same chemical species may have multiple catalytic sites, and that in different reactions the activity of the same site may be influenced by the context. In this paper, however, we ignore such complications, in order to investigate the effect of the introduction of the pure structural constraint on the final organization of the chemistry thus constructed.

The model allows for calibrating catalyst specificity, an important aspect in origin-of-life scenarios. In current systems, after billions of years of evolution, enzymes are extremely powerful and specific, capable of accelerating the rate of a single reaction millions of times compared to the uncatalyzed reaction [29,30]. This high specificity is captured by Kauffman’s original model, in which each catalyst typically catalyzes only one reaction, or in rare cases a few reactions. In early prebiotic systems, however, catalyst specificity is thought to have been limited [31,32]; often, the same catalyst was able to accelerate—possibly only slightly—a large set of reactions. In the C-BPM model it is possible to adjust the degree of specificity of the reactions by modifying the length of the active site.

Both in the K-BPM and in the C-BPM case it is possible to generate the total chemistry adopting the approach that considers the total number of possible species, or to explore the chemistry starting from a firing disk.

**Figure 2:**
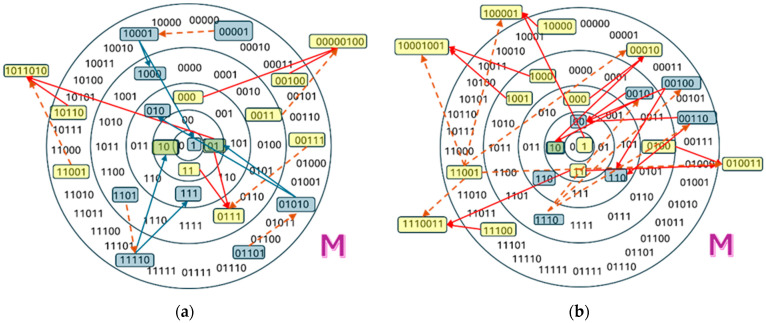
Panels (**a**,**b**) show the initial stages in assembling K-chemistry and C-chemistry, starting from chemical species whose maximum length is *M*. Dashed brown arrows mark catalytic relationships (which catalyst drives the production of which chemical species). Production relationships are shown with solid blue arrows for cleavage processes and solid red arrows for condensation processes. In the K-chemistry depicted on the left, most reactions rely on distinct catalysts whose structures bear no resemblance to those of the corresponding reactants. On the rare occasions—purely accidental—a single catalyst promotes multiple reactions; the reactions themselves share no structural connection. Condensation steps may produce molecules longer than any of the original species (exceeding the “firing disk” radius). The final situation is such that a large number of reactions requires a large number of catalysts. In the C-chemistry on the right, each catalyst typically catalyzes more than one reaction, all triggered through the same active site. Consequently, only a small set of catalysts (in the figure, only the species 11001 and 1110) is needed to support a large number of reactions.

In the case of a firing disk, a small number of randomly selected initial polymers are assumed to be cleavage or condensation catalysts (see Figure 2). The initial species are analyzed for complementary matches; when one is found, the corresponding reaction occurs, which can generate new species. Each time a long enough new polymer is generated, it is randomly chosen as catalyst with a small, fixed probability *p_cat_*. The reactions that this polymer can catalyze are then searched for. The process is iterated until there are no more new reactions or new species to add, or some termination condition is met. Table 1 summarizes the main parameters of the model, and those concerning the initial setting.

### 2.3. Characteristics of the C-Chemistries

In the case of systems with firing disks of the same size, small values of *p_cat_* lead to small chemistries, without autocatalytic sets, and larger values of *p_cat_* lead to large chemistries, all with an autocatalytic set. If we assume that no species larger than *L_max_* can be created (thus also limiting the maximum number of species), one can observe that the large chemistries tend to generate almost all possible chemical species, achieving very high connectivity (defined here as the number of reactions divided by the number of species) and hosting a RAF often as large as the entire chemistry, while less than 4% of small chemistries contain a RAF [27]. Conversely, for the same *p_cat_*, increasing the number of initial species in the firing disk leads more easily to the creation of large-scale chemistries. In fact, a similar dependence (on the size of the firing disk, and on the probability of creating reactions) had also been reported for K-chemistries [14].

**Figure 3:**
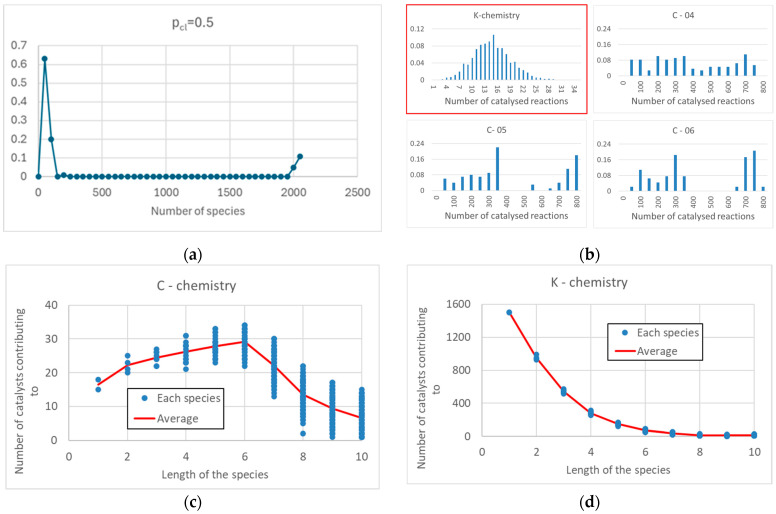
Set of measurements performed on an ensemble of 100 networks, with *p_cat_* = 0.05, *p_cl_* = 0.5, 24 initial species of maximum length equal to 4, *L_max_* = 10, size of the active sites uniformly distributed between 3 and 4. (**a**) Size distribution of C-chemistries: two groups of large and small chemistries can be identified, with no intermediate-sized instances. (**b**) Some examples of the distribution of the number of reactions catalyzed by each individual catalyst (in three particular networks extracted from the previous ensemble, bin size = 50), compared to the same distribution in a typical K-chemistry (highlighted with a red outline) having the same number of species and reactions (bin size = 1). (**c**) The plot shows in a typical C-chemistry the number of catalysts contributing to the formation of a particular species, as a function of the length of the species—in this network there are only 100 catalysts. (**d**) The same as in (**c**), for the K-chemistry present in (**b**); we can note the very high number of catalysts and the fact that, at any length, the variability in the C-chemistry is greater than that in the K-chemistry.

A large C-chemistry has a high number of reactions, despite the presence of a low number of catalysts. To better define the meaning of “low” and “high,” however, a benchmark is needed. A good reference is the well-known K-BPM model, which reflects the idea of high catalytic specificity combined with a lack of relationship between the structure of catalyst and reactants. To achieve connectivity levels comparable to those observed in the large C-chemistries of Figure 3 (typically 2000 species and 40,000 reactions), the species in a K-chemistry having the same number of species and reactions would have to catalyze on average about 20 reactions. Moreover, in any C-chemistry the number of reactions catalyzed by each catalyst is significantly higher than the number of reactions catalyzed by each catalyst in a K-chemistry (Figure 3b).

The distribution of the number of reactions catalyzed by each catalyst in a K-chemistry takes the form of a bell curve, while in a C-chemistry it takes an irregular and wider shape (Figure 3b). This is indeed a significant difference between the two models, and it might be interesting to see clues of this observable in real systems. We will make comments on this in the final section. In C-chemistries, the average number of reactions catalyzed by each catalyst (~400 reactions) is much higher than the average number of species catalyzed by it (210 species)—the opposite situation to that in K-chemistries, where the average number of reactions catalyzed by each catalyst (~20 reactions) is lower than the average number of species catalyzed by it (26.8 species). In both cases, there are different reactions able to produce the same chemical species. The number of different catalysts that lead to the synthesis of the same product (i.e., the number of different pathways) is shown in Figure 3c,d as a function of the length of the product. There are many more catalysts in the case of K-chemistry, and the trends are very different the ways to obtain the same product are a decreasing function of its length in the K-case, while there is a maximum value for C-chemistries.

### 2.4. Emergence of Collectively Autocatalytic Systems

The C-BPM model involves several parameters, and a large fraction of implementations leads to poor chemistries of little interest. In order to focus computational resources on significant areas, an alternative strategy can usefully be adopted to determine whether a particular chemical reaction system is formally favorable to the formation of autocatalytic sets. This approach assumes that all species that can be built are potentially present and gradually adds chemical reactions, monitoring the presence of RAFs. In C-chemistries this means identifying an increasing number of catalysts, and in K-chemistries this means adding an increasing number of chemical reactions (each one requiring a catalyst).

In the following simulations, we will use systems composed of 510 chemical species (all polymers up to 8 monomers long), including 14 food species (polymers up to 3 monomers long). We assume that the catalyzing species are at least 4 monomers long (in C-chemicals, the active site can be 4 or 5 monomers long). For each number of catalysts or reactions added, 20 different random realizations are analyzed.

**Figure 4:**
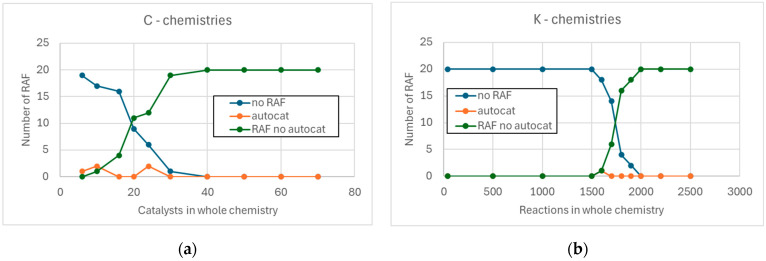
Number of RAFs composed of a single catalyst (“autocat”) and other RAFs. (**a**) C-chemistries, as the number of added catalysts varies. (**b**) K-chemistries, as the number of added chemical reactions varies.

As the number of reactions added in both chemistries increases, the number of RAFs present in the various realizations increases, until saturation is reached (all realized chemistries contain a RAF). However, the number of RAFs in the C-chemistries initially shows a linear trend, while the K-chemistries remain without RAFs for a long time, and then saturate very quickly (see Figure 4). Indeed, this abrupt change is reminiscent of a phase transition.

**Figure 5:**
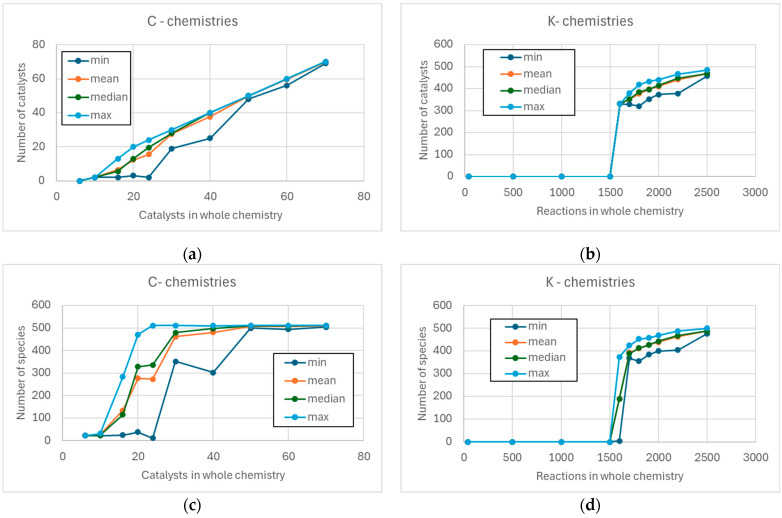
In the left column are present the C-chemistries, in the right column the K-chemistries. (**a**,**b**) Number of reactions (minimum, mean, median, and maximum) in an RAF in C-chemistries and (**b**) in K-chemistries. (**c**) Number of chemical species (minimum, mean, median, and maximum) in a RAF in C-chemistries and (**d**) in K-chemistries.

In fact, while in C-chemistries many indicators (number of catalysts present in a RAF, fraction of chemical species in the RAF that are catalysts) continue to vary well beyond the moment in which the number of found RAFs reaches saturation, in K-chemistries these indices remain equal to zero until almost the critical threshold (located between 1500 and 1600 reactions), and then they suddenly reach their maximum values (Figure 5a–d). In C-chemistries the number of species belonging to a RAF shows a sigmoidal trend—but this is essentially because the graph saturates when the maximum number of species is reached (Figure 5c).

In C-chemistries only the number of species belonging to a RAF shows a sigmoidal trend (Figure 5c), but this is simply due to the limited maximum size of the systems analyzed here. In K-chemistries, therefore, only two typical conditions exist: the absence of RAFs or the presence of RAFs. When RAFs are present, they are immediately large (Figure 5b,d), and most of the chemical species involved are also catalysts. This combination of characteristics makes the RAFs very difficult to dynamically sustain, as observed in several works [3,16,27].

C-chemistries, on the other hand, can host RAFs even when the number of active catalysts is very low (a feature absent in the case of K-chemistries). As long as the number of catalysts is small, these RAFs are typically small, and only a small fraction of the involved chemical species are also catalysts—a feature present also in the case of large RAFs. As observed in this work, this combination of characteristics makes the RAFs of C-chemistries likely to be dynamically sustainable.

**Figure 6:**
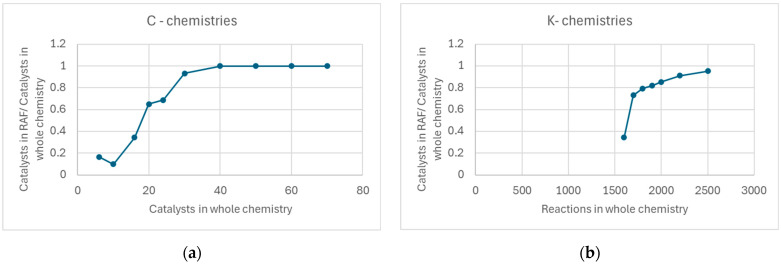
Number of catalysts in a RAF divided by the number of catalysts in the total chemistry (medians only). (**a**) C-chemistry. (**b**) K-chemistry—in this situation, the first RAFs are present only after the introduction of at least 1600 reactions.

It is interesting to observe the fraction of catalysts involved in a RAF compared to the total number of catalysts present in the system. Again, in the case of K-chemistries there is a sharp variation, and typically this fraction is very high (the chemically active universes are, so to speak, “permeated” by a RAF), while in the case of C-chemistries, it is possible that large parts of the world do not host any autocatalytic systems—Figure 6.

### 2.5. Correlations Among Components

One of the differences between the RAF construction process in C-chemistries and K-chemistries is that in the former case many reactions share common cores (the complement of the active site if they have the same catalyst), and consequently, the structures of many condensation products should include similar components. Cleavage action also has similar characteristics. As we will see below, this gives rise to correlations between the structures of the species belonging to the RAF, different from that existing in random collections of species extracted from the chemistry to which the RAF belongs.

**Figure 7:**
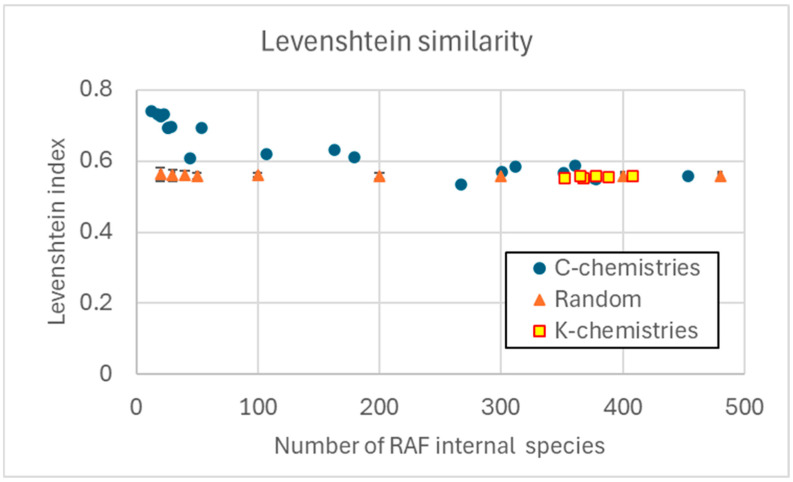
Levenshtein index, as the number of chemical species present in a RAF varies, excluding food species. Circles indicate the index value in RAFs present in the C-chemistries with 60 and 100 catalysts; squares indicate the index value in RAFs present in some K-chemistries. Triangles indicate the index value in random extractions of species from the entire world (excluding food species); each symbol represents the average of 100 different random extractions. Also included are the bars corresponding to the standard deviations, which are so small that they are often obscured by the triangular symbols.

It is interesting to observe whether a similar situation exists in RAFs belonging to K-chemistries, in which the relationship between catalyst and reaction is not based on structure. There are many ways to quantify relationships between strings in a set. Here we will use the Levenshtein distance [33], which measures the similarity of two strings on the basis of the minimum number of character changes required to transform one into the other, normalized with respect to the maximum size of the two strings. The normalized Levenshtein similarity (NLS) is the one’s complement of this normalized distance (0.0 indicates completely different strings, and 1.0 identical strings); applied to a set of strings, it can form a pairwise similarity matrix. We show in Figure 7 the average values of the terms outside the main diagonal of these large matrices (obviously excluding the comparisons of each string with itself).

Specifically, we are interested in the species belonging to the RAFs of the C-chemistries and the RAFs of the K-chemistries; from these sets, we remove the strings corresponding to the food species, which are the same in all sets. We then use sets of the same size, randomly drawn from universes including the RAFs (again excluding the food species) as control groups.

As already noted, in Figure 7 it is possible to observe that in K-chemistries there are no RAFs with fewer than 300 chemical species: the similarity measures of such sets are almost identical to those of random extractions from the universes they belong to. The similarity of RAFs of C-chemistries, however, shows different values, increasingly shifting towards high values as the size of the RAFs themselves decreases. In RAFs whose dimensions are comparable to those of the host C-chemistries, these values are not too far from those of the host universes, while in RAFs that are much smaller than the host universes, the values are many standard deviations larger than the averages of the corresponding random draws.

## 3. Dynamics

In this work, we are interested in autocatalytic sets in protocells, capable of self-sustaining and of reproducing, giving birth to other similar entities. To achieve and maintain these characteristics, a protocell must (i) produce its internal materials, (ii) produce the lipids for its container, (iii) in sufficient quantities to grow and then divide into two parts. Although no viable protocells have yet been observed, they are thought to have been crucial to the self-organizing processes that gave rise to life. Moreover, studying them may be essential for developing new forms of “protolife” capable of adaptation and evolution.

Different protocell architectures have been proposed [34,35,36,37,38,39,40,41,42]. The model we consider here includes the presence of a semipermeable membrane, which can be crossed by simple chemical species (assumed to be present in the environment) via passive diffusion. These species can be used by catalyzed reactions to produce other, more complex chemical species, no longer able to cross the membrane; some of these species could catalyze chemical reactions. If the chemical arrangement is such as forming a RAF (in which the simple chemical species provided by the environment play the role of food), it may happen that the chemical reactions support the dynamic reproduction of internal materials.

**Figure 8:**
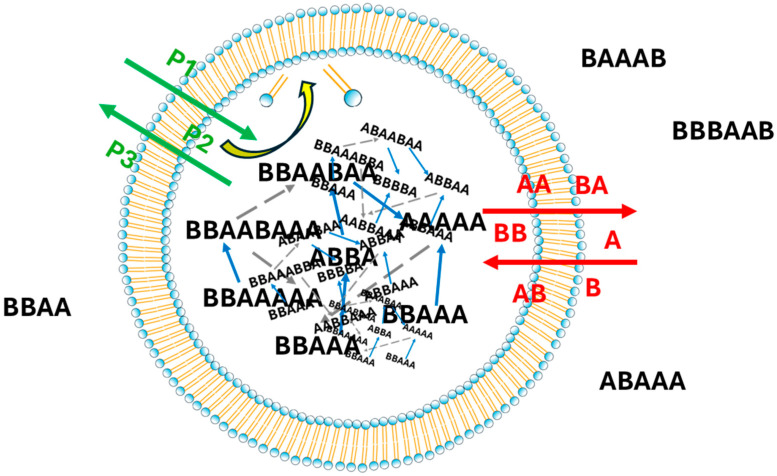
Protocell diagram. Inside the membrane, there are chemical species linked by relationships of production or catalysis. Some of these species can cross the membrane (the species near the arrows that cross the membrane): the “Food” species are highlighted in red, and the membrane precursors (P1, P2, etc.) in green. The precursors are reactants in reactions (catalyzed by some of the internal species) able of directly producing membrane materials, which are assumed to be quickly incorporated into the membrane itself (the yellow arrow).

However, the growth of the internal materials must be coordinated with the growth of the container. For this purpose, we assume that a number of *N_catM_* chemical species (not belonging to the Food) of the RAF are able to catalyze some reactions that, using simple precursors (independent of the Food of the RAF, but similarly capable of crossing the membrane), produce the lipid constituting the container; the lipid produced in this way is assumed to be instantly integrated in the membrane (see also Figure 8). When the membrane reaches a critical size, it divides in two, thus creating two protocells smaller than the original one. The division process is assumed to be fast enough not to significantly change the concentrations of the internal materials.

We have shown in [12] that in general there are only two possible final outcomes of such a reproduction mechanism: sustainable growth (“synchronization” of the growth rates of the internal materials and the container) or starvation (the internal material is increasingly diluted, causing increasingly longer duplication times, until growth essentially ceases).

The model requires the determination of a large number of parameters. A possible starting point is to assign the same value to the kinetic coefficients of all reactions, then to introduce randomness and possibly to include a bias favoring condensations or cleavages. Most of the results of this work are based on the first approach, while in selected cases we have also observed the contribution of randomness and bias. It is then necessary to determine the kinetic coefficients of the reactions capable of producing lipids, the membrane diffusion constants of the substances capable of crossing the container, and their external concentrations (assumed to be constant in this work). We will assume that each of the reactions capable of creating lipids (catalyzed by different chemicals belonging to the RAF) uses a different precursor (however, some simulations were performed in which there was a single precursor, without noticing qualitatively different results). Table 2 summarizes the values of the main parameters used here.

Interestingly, the presence of RAFs does not guarantee synchronization. Of course, it is possible to design RAFs that support synchronization [3,12]; often such RAFs are small, but it is possible to envision even large RAFs. For example, an (ordered) set of chemical species in which every *i*-th chemical catalyzes the production of the (*i* + 1)-th, in which the last species catalyzes the production of the first (thus forming a large cycle), and in which each reaction uses only food species as reagents, has a high probability of allowing protocell synchronization, if one or more of its own species also catalyzes the growth of the container. Such an organization, however, has an extremely low probability of spontaneously occurring in a prebiotic environment.

The question is therefore whether randomly organized chemical species can create RAFs capable of sustaining the synchronization of a protocell. As anticipated, the question can be split into two: which general chemical organizations can host which types of RAFs, and which types of RAFs are capable of sustaining the dynamics of a protocell.

In the previous section, we showed that K-chemistries exhibit a sharp transition as the number of possible reactions increases; below a certain threshold, there are no RAFs, while above it, the presence of a RAF is almost certain. In this case, the RAF is large, typically as large as the chemistry hosting it. Previous work suggests [3], and current work (with a more sophisticated protocell model) confirms [16,27], that protocells containing large RAFs typically do not synchronize. In particular, none of the RAFs we found in K-chemistries, if embedded in a protocell, allow synchronization. These observations should be contrasted with the behaviors found in C-chemistries, where a significant fraction of the RAFs lead to the formation of synchronizing protocells.

It should be noted that in the following, we consider a protocell to be “synchronizing” if we achieve convergence to a finite duplication time (with a maximum set below at 150,000 time units) in a finite number of generations (30 generations), on the first attempt, without varying the value of the kinetic coefficients (see Table 2). Variations in the indicated hyperparameters do not lead to qualitative changes in the presented trends. Finally, let us assume that there are five types of membrane precursors, catalyzed by five chemical species belonging to the RAF (we will present the consequences of this choice below). Note that the probability of formation of synchronizing protocells in C-chemistries is much higher than the same probability in K-chemistries.

The phenomenon is due to two interesting factors: (i) C-chemistries can present small-sized RAFs (a situation that very often allows protocell synchronization) and (ii) quite often also large-sized RAFs allow synchronization in C-chemistries.

**Figure 9:**
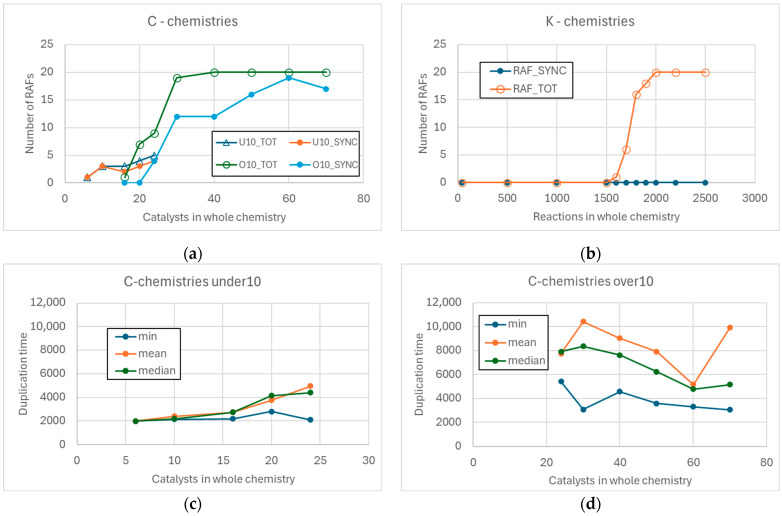
(**a**) Number of RAFs and of synchronizing RAFs (out of a total of 20 C-chemistries) comprising up to 10 catalysts, and with more than 10 catalysts, as the number of catalysts in the hosting C-chemistry varies. (**b**) Number of RAFs and of synchronizing RAFs (out of a total of 20 K-chemistries), as the number of catalysts in the hosting K-chemistry varies—all the RAFs present are large RAFs. (**c**) The minimum, mean, and median duplication times of synchronizing protocells when supported by RAFs of up to 10 catalysts. (**d**) The minimum, mean, and median doubling times of synchronizing protocells when supported by RAFs with more than 10 catalysts. In small RAFs, it is possible to note a slight increase in the duplication times as the number of catalysts (and chemical species) present increases, while in the case of large RAFs the variability covers a trend that appears very flat.

In Figure 9, it can be observed that well over 60% of small RAFs synchronize, while larger RAFs when present in chemistries of the same size are unable to do so (Figure 9a). It can be noted that under similar low-connectivity conditions the K-chemistries do not exhibit any RAF. Large RAFs become able to synchronize only when the number of catalysts is sufficient to significantly increase the number of RAF reactions; 60% of them synchronize in C-chemistries containing up to 40 active catalysts, and over 80% if more catalysts are present (Figure 9a).

The ability to synchronize therefore appears to be a fairly common feature in RAFs embedded in C-chemistries. However, it is important to evaluate the contribution to this feature of the characteristics of the list of species identified as catalysts for reactions capable of producing lipids (in short, “membrane catalysts”) and therefore for the growth of the container. Indeed, the details of the species, and/or their number, can be varied in different simulations. In K-chemistries, RAFs are always large in size, and we have never seen synchronization (Figure 9b).

We then performed experiments on some of the previous chemistries, where we varied the number of membrane catalysts (from just 1 up to the 5 of the previous experiments), in 20 different sets for each number of catalysts.

**Figure 10:**
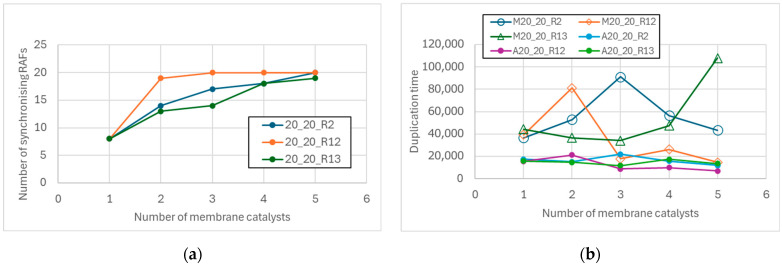
(**a**) Number of synchronizing protocells (out of 20 different choices of membrane catalysts) as the number of membrane catalysts varies, in three different systems embedded in chemistries with 20 cleavage catalysts and 20 membrane catalysts. (**b**) Average (A_*xx*) and maximum (M_*xx*) duplication times of the synchronizing systems as the number of membrane catalysts varies.

The results show (see Figure 10 for the details of some of the chemistries we analyzed) that, as can be expected, different choices of membrane catalysts can lead to different outcomes (e.g., different duplication times, up to the absence of synchronization). Moreover, it is interesting to observe that the lower the number of membrane catalysts, the lower the probability of synchronization.

It is noteworthy that these effects are obtained despite the fact that the different selections of membrane catalysts do not lead to changes in the RAF itself. Membrane catalysts are, in fact, entities normally participating to the RAF, and—given the nature of the catalytic action—their concentration is not modified by their action on membrane reactions. By design, the flux of membrane precursors does not interfere with, nor is it influenced by, the flux of food species. So, a different choice of membrane catalysts can only lead to a change in the duplication times of the protocells. Interestingly, this difference in dilution factor (i.e., the growth rate of the container) can lead to dramatic changes in the concentration profiles of all the chemical species belonging to the RAF.

It is possible to describe the feedback at work. If the concentrations of the membrane catalyst are high, lipid production is fast, and the protocell replicates rapidly. If these concentrations are low, the replication time is very long; if they are very low, the protocell even fails to replicate. What determines the concentrations of the membrane catalyst—in addition to the dilution factor—is the quantity of those that are produced by the reactions of the RAF to which they belong; the rate of these reactions, in turn, is determined in a nonlinear manner by the concentration profile of the species (reactants and catalysts) that are present in the RAF itself. The concentration profile depends on the dilution rate. At the end the system reaches a steady state, or a starvation condition, where the concentration of membrane catalysts is minimal and the absence of growth of the container leads to the uniformity of the internal and external concentrations of food species.

The dynamics of this feedback, however, is actually very complex. One example can show how difficult it may be to determine which chemical species can be effectively selected to enable protocell synchronization. Analyzing a non-synchronizing protocell, one always notices that the concentrations of membrane catalysts are extremely low, while other chemical species may be present at very high concentrations. If the simulation is repeated, selecting these latter species as membrane catalysts, their production could be expected to be high enough to allow them to support stable replication. The typical outcome, however, is another non-synchronizing case, with completely different concentration profiles—and with concentrations of these membrane catalysts at extremely low levels (notwithstanding their previous very high concentrations).

It is possible to give more quantitative indications by considering a particular system (named M12_12_R18_5cat), a realization of a C-chemistry containing a RAF achieved via 12 cleavages and 12 condensations, and 5 membrane catalysts. This is a typical system, whose behavior is similar to the other synchronizing systems in this work.

**Figure 11:**
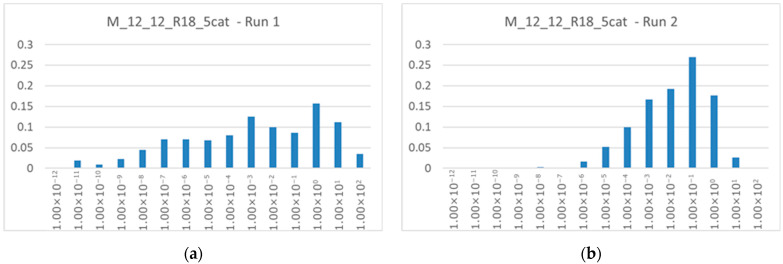
Distribution of non-food chemical species concentrations of the RAF present in the M12_12_R18_5cat system (14 foods, 421 internal chemical species, 2022 reactions, 5 membrane catalysts). (**a**) First configuration of membrane catalysts, doubling time 76,330. (**b**) Second configuration of membrane catalysts, doubling time 6160. It can be noted that a high doubling time allows at the same time the presence of species at very low concentrations (less than 1 × 10^−6^) and very high concentrations (greater than 1 × 10^1^), more than a lower doubling time does.

**Figure 12:**
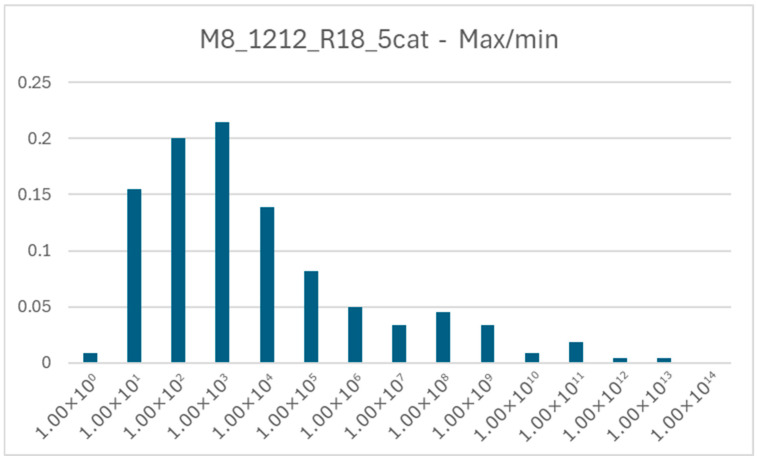
Distribution of the ratio between the maximum and minimum values of concentration for each species in the case of the M12_12_R18_5cat system. It is possible to see that a single species can be present in different runs with even very heterogeneous concentrations.

In general, the longer the duplication time, the higher the concentrations of internal chemical species. Considering each concentration as a component of an N-dimensional vector (with N equal to the number of chemical species considered), the magnitude of this vector scales linearly with the doubling time. In the case of low duplication times, the concentration profiles are narrower and sharper (Figure 11). We can add that, out of the 421 internal chemical species of the system under consideration, 305 have non-zero concentrations in every run, 109 appear in fewer than seven runs (of which 23 appear in fewer than five), and none is always absent. Therefore, there is a “hard core” of species that are always present, and a halo of species that are not always present. A single chemical species can be present in the twenty simulations with concentrations that differ greatly, even by many orders of magnitude. Figure 12 shows the distribution of the ratio between the maximum and minimum values of each species in the M12_12_R18_5cat system; in these simulations, the ratios are typically around 10^3^ but some can exceed 10^12^.

**Figure 13:**
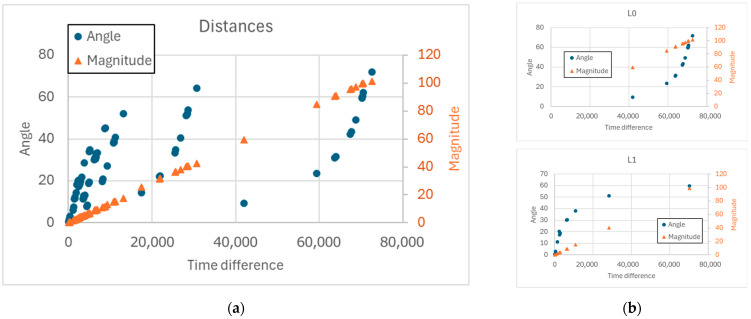
(**a**) Trend of the distances (in angular or magnitude terms) versus the difference in duplication times, for all possible pairs in the case of the M12_12_R18_5cat system. (**b**) Detail for the pairs formed by the first and second launch, and by the first and third launch. In (**a**), it is possible to notice that similar time differences can correspond to angles (and therefore compositions) that are sometimes very different. If we use a single run as a reference—part (**b**) of the figure—we can note the kinds of correlation between duplication time differences and angles and distances between compositions.

If we compare pairs of different runs, it is possible to observe that the greater the distance between the doubling times, the greater the difference between the concentration profiles of the species belonging to the RAF (whose structure is the same in all the runs). This difference can be measured in terms of the angular distance between the vectors representing two concentration profiles or as the magnitude of their difference. In each observed pair both variables increase as the difference in doubling times increases (Figure 13).

**Figure 14:**
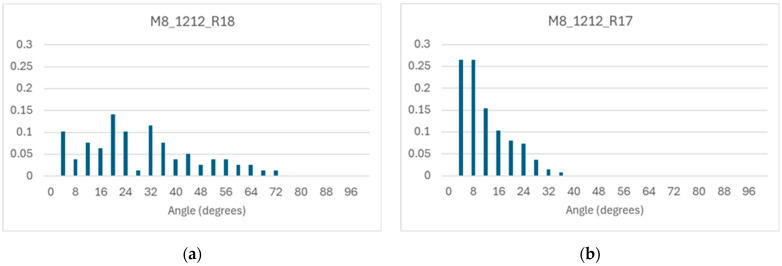
Distribution of angle among different runs, in different systems. (**a**) M12_12_R18_5cat system. (**b**) M12_12_R17_5cat system (another realization of a C-chemistry containing a RAF achieved via 12 cleavages and 12 condensations, and 5 membrane catalysts). Low angles indicate similarity of composition; angles close to 45° indicate no correlation. In one system this value is well within the distribution, in the other no pair reaches this value; different RAF structures can therefore exhibit different levels of flexibility.

The distributions of angles between pairs of runs (which differ only in the choice of catalysts) can be different in different systems (which differ in chemical reaction topology); Figure 14 shows two of these distributions, highlighting that different topologies can have different behaviors in response to changes in membrane catalysts.

In summary, focusing on conditions where synchronization is relatively frequent (using five membrane catalysts), it is possible to observe that the structure of a RAF is important:

- RAFs that allow a protocell to synchronize tend to do so even when varying the composition of the membrane catalysts (Figure 9);
- RAFs that do not allow a protocell to synchronize tend not to synchronize even when varying the composition of the membrane catalysts (none of the non-synchronizing RAFs in Figure 9 are able to synchronize, even when varying the composition of the membrane catalysts.

Simply varying the list of membrane catalysts can significantly change both the doubling time and the steady-state chemical composition. However, once again, the structure of a RAF plays a significant role; in some RAFs, this variation leads to very heterogeneous compositions, while other RAFs are able to maintain more homogeneous chemical compositions (Figure 14).

## 4. Conclusions

Our main motivation for this study, as discussed in Section 1, is the interest in the origin of life, and in the importance of polymers in this process. We have relied on a well-known abstract polymer model, the K-BPM, and we have also proposed and analyzed a modified version, the C-BPM, which captures a relationship between the structure of a polymer and its catalytic properties—a relationship that is absent in the K-BPM.

The OOL is a complex, multi-step process, which cannot be fully described with stylized models like the BPMs, but we studied their behaviors to get insights about the way in which life might have emerged, on the Earth or elsewhere, focusing on catalyzed molecular replication and on protocell reproduction. RAF sets are useful graph-theoretical tools for identifying potentially interesting structures.

The analogue of OOL, in these stylized models, is the sustainable growth of a population of protocells. A major result of the studies, described in Section 2 and Section 3, is that the presence of a RAF set does not imply synchronization, which is a necessary condition for the existence of a sustainable population of protocells. In brief, RAFs do not necessarily imply life. But this is not the whole story. Actually, in K-chemistries one can find only large RAFs (as can be argued by the sharp transition in RAF size as the number of reactions grows—see Figure 5), and there is no synchronization. But in C-chemistries the size of RAFs grows smoothly, and for a wide set of values one can also find small RAFs, which are usually also accompanied by synchronization. Moreover, in C-chemistries large RAFs can also synchronize. These differences highlight the importance of C-chemistries.

The fact that in K-chemistries large RAFs inhibit synchronization had also been previously observed [3,27]) and it is likely related to the presence of several competing reactions, which are infrequent in small RAFs. This suggests that in these systems synchronization may be more easily achieved if the overall network is divided in modules, which can be separated in two not mutually exclusive ways, i.e., either physically (e.g., by embedding some subnetwork in organelles) or functionally, by limiting the number of connections between different subnetworks. It should also be interesting to consider how modules change during the growth of the overall network, under the action of evolution; this of course requires the development of a suitable model of the way in which these systems undergo evolution. The same type of analysis would be interesting also in the case of C-chemistries.

An interesting feature introduced by the dependence of catalytic activity on the structure of the molecular types participating in the reaction appears to be a change in the distribution of the number of reactions catalyzed by each catalyst, compared to a situation where this dependence is absent. It could be interesting to have clues to a similar observable in real systems. The enzymes currently present in cells appear to be highly specific, catalyzing a single reaction, but they are the result of billions of years of evolution. In a pre-biological context, the situation could have been very different, and some clues could be found in non-biological organic chemical systems, e.g., underwater hydrothermal vents [43,44], terrestrial hot springs [45], or reconstructions of primitive Earth conditions [46,47,48].

It would also be worthwhile, in future works, to analyze the behavior of different models, inspired by the present one, which overcome some of its limitations.

First, one could consider systems of longer polymers, whose catalysts can host longer active sites that endow them with higher specificity. These modifications would allow not only a thorough exploration of the behavior of systems of different specificity, but also the analysis of the effects (if any) related to the co-existence of more and less specific catalysts. Note, however, that the use of longer polymers implies an (exponential) increase in the number of possible different species, and therefore a heavier computational load.

Another possible generalization of the models described here would be that of using a discrete “alphabet” with more than two “letters”, i.e., with more than two types of different building blocks, which allows the hosting of a larger number of different polymer species in sequences of a given length. If one wants to keep a complementary matching rule, it would then also be necessary to suitably define complementarities (e.g., the pairs A-T and C-G in DNA). Using nonbinary vocabularies would of course shed light on the possible effects of the size of this basic vocabulary on the overall system behavior.

Further limitations of the present models are related to the use of unidirectional reactions. While chemical reactions are of course bidirectional, this approximation is justified when the difference between the free energy of the products and that of the reactants is high. Actually, the rate of the reverse reaction can become comparable with the rate of the forward reaction when the concentration of the reactants is extremely low and that of the products is extremely high, but this happens in quite extreme cases. When the reverse reactions must be taken into account (see, e.g., [19,49]), one typically observes an increase in the number of autocatalytic sets, while simulations are required to unfold the effects on the dynamics of these chemical reaction systems.

One could explore the possible effects of other features that have been overlooked so far, including, e.g., partial matches, nonlinear polymers, non-catalyzed reactions, and others. However, a word of caution: making a model more complicated is usually an easy exercise, which should be limited to modifications that are perceived as really necessary (like the link between the sequence and the catalytic activity explored in this paper) or that allow one to ask new interesting questions.

Note that the results presented here also raise foundational questions about the effectiveness of continuous models (like the so-called law of mass action) in describing chemical reactions. Time-continuous models imply the need to take a limit *Δt*→0, but chemical reactions involve molecular encounters, which are discrete events. If there are several specimens of each molecular type, then taking the limit, as is usually done in chemical kinetics, makes sense and is effective, while it becomes questionable when some molecular types may be present at very low concentrations. The present paper shows that the concept of RAF, as presently defined, is not well-suited to deal with cases where some types of chemicals may appear or disappear. In these cases, using time-discrete models (e.g., cellular automata or the Gillespie algorithm) may turn out to be necessary, but this would require a revised definition of RAF, if its presence should imply an autocatalytic behavior. We limit here the mention of these problems, as indication for future works, which lie beyond the purpose of the present one.

## Figures and Tables

**Table 1:** The table summarizes the main parameters of the C-BPM model, including those concerning the setting of the firing disk.

Model	Symbol	Description
Firing disk	*FD_Lmax_*	Maximum length of species present in the firing disk
	*NCL_ini_*	Number of cleavage catalysts present in the firing disk
	*NCD_ini_*	Number of condensation catalysts present in the firing disk
C-chemistry	*L_max_*	Maximum length of species belonging to chemistry
	*L_smin_*, *L_smax_*	Minimum and maximum length of the active site
	*p_cat_*	Probability that a new chemical species is a catalyst
	*p_cl_*	Probability that a catalyst is catalyzing a cleavage

**Table 2:** The table reports the values of the coefficients used in simulations (see Appendix B for a more detailed description). Other series of simulations, in which the values of the kinetic coefficients of each individual reaction (be it cleavage, condensation, or a reaction capable of producing membrane materials) were randomly varied by several orders of magnitude, showing trends qualitatively consistent with those shown in this paper. The same is true by varying the diffusion constants across the membrane and/or the external concentrations of food and membrane precursors.

Coefficient Name	Typical Value
Kinetic coefficients of cleavages	0.01 M^−1^ s^−1^
Kinetic coefficients of condensations	0.01 M^−2^ s^−1^
Kinetic coefficients of membrane reactions	0.01 M^−1^ s^−1^
Membrane diffusion constants (Food)	1 × 10^−18^ cm^2^ s^−1^
Membrane diffusion constants (membrane precursors)	1 × 10^−18^ cm^2^ s^−1^
External concentrations of Food	10 M
External concentrations of membrane precursors	10 M

## Data Availability

Dataset available on request from the authors.

## Appendix A

Steel and Hordijk [25,50] proposed the concept of Reflexively Autocatalytic Food-generated set (shortly RAF set, or simply RAF), which is useful to identify systems potentially able to support (i) the reproduction of catalysts and (ii) the reproduction of the necessary substrates (starting from the food, i.e., a set of chemicals provided by the environment). A precise definition of a RAF, following [25,51], is given below.

A catalytic reaction system (CRS) over a food source ***F*** is defined by a triplet ***L*** = (***X***; ***R***; ***C***) where ***X*** is the universe of all possible molecular types (that includes catalysts and substrates), ***R*** is the set of all the reactions that can occur among these molecules and ***C*** is the set of all the pairs (*x*, *r*) where *x* ∈ ***X*** and *r* ∈ ***R*** and *x* catalyzes *r*. ***F*** is a subset of molecular types (***F*** ⊂ ***X***) that are supplied from the outside and are available even if the system ***L*** is unable to synthesize them.

In a catalytic reaction system ***L***, a Reflexively Autocatalytic Food-generated set is then defined as a subset ***R*′** ⊆ ***R*** of all possible reactions that is:

1. Reflexively autocatalytic (RA): each reaction *r* ∈ ***R*′** is catalyzed by at least one molecular type belonging to ***L***;
2. Food-generated (F): all the chemical species in ***L*** that do not belong to the food set ***F*** can be synthesized from ***F*** by using only reactions in ***R**′***.

So, a set of reactions ***R**′*** is a RAF:

- if each reaction is catalyzed by one or more chemical species involved in a reaction in ***R**′***;
- and if each reactant in ***R**′*** can be built starting from the food set ***F*** by successive applications of reactions from ***R**′***.

These rules capture, on a static structure such as a reaction graph, the abstract idea of “life” as an auto-catalyzing system able to maintain itself by using a suitable food source [25]. Dynamical effects (e.g., an exceedingly slow reaction rate) might of course affect the growth rate.

The definition implies that, if there is a RAF and if no species in ***F*** is a catalyst, then a cycle of autocatalytic reactions is necessarily a subset of the RAF; otherwise, it would be impossible to satisfy the condition that all the reactions are catalyzed by at least one species in ***R**′***. However, if the food set contains catalysts, then the RAF property might be satisfied also by linear reaction chains, having their “roots” in ***F***.

A RAF has been defined as a set of reactions, but of course the definition makes sense in a particular catalytic reaction system ***L***, so the chemical species are also necessary to guarantee the RAF property of a set of reactions. Moreover, since in different models of random “chemistries” the same reaction can be coupled to different catalysts, also the set C of pairs {reaction, catalyst} has to be implicitly assumed. In this paper, when no ambiguity is possible, we will sometimes take the liberty of referring to the “species belonging to a RAF”.

## Appendix B

Here we present the diagram of a simple protocell, to introduce the mathematical formalism and the coefficients used. Suppose the significant reactions are:

(A1) $$\begin{array}{c} P+X\to C+X\\ F+X\to X+X\; \end{array}$$

In this system, *X* is a substance that can catalyze its own production using the Food *F* and can catalyze the production of the membrane *C* starting from the precursor *P* (note that in this simplified example the reactions are simple transformations, from *P* to *C* and from *F* to *X*). To model the reactions, we will use the law of mass action. Substances *F* and *P* can cross the membrane via passive transport, modeled by the Fick equation [52,53].

(A2) $$\left\{\begin{array}{c} \frac{dC}{dt}=\alpha V\left[X\right]\left[P\right]\\ \frac{dX}{dt}=\eta V\left[X\right]\left[F\right]\\ \frac{dF}{dt}=\frac{D_{F}SK\left(F^{*}-\left[F\right]\right)}{\delta }-\eta V\left[X\right]\left[F\right]\\ \frac{dP}{dt}=\frac{D_{P}SK\left(P^{*}-\left[P\right]\right)}{\delta }-\alpha V\left[X\right]\left[P\right] \end{array}\right.$$

where *S* and *V* indicate the surface area and the volume of the protocell, *F** and *P** are the external concentrations of Food and membrane precursor, and *δ* indicates the membrane thickness.

Let us make the concentrations explicit, so as to use only quantities:

(A3) $$\left\{\begin{array}{c} \frac{dC}{dt}=\alpha \frac{XP}{V}\\ \frac{dX}{dt}=\eta \frac{XF}{V}\\ \frac{dF}{dt}=\frac{D_{F}K}{\delta }S\left(F^{*}-\frac{F}{V}\right)-\eta \frac{XF}{V}\\ \frac{dP}{dt}=\frac{D_{P}K}{\delta }S\left(P^{*}-\frac{P}{V}\right)-\alpha \frac{XP}{V} \end{array}\right.$$

Assuming that the protocell is approximately circular, we can express *S* and *V* as a function of the quantity of membrane material *C* alone (*S = C*/*(ρδ*), with *ρ* equal to the density of the material *C*, and $\chi =\frac{1}{6\delta^{\frac{3}{2}}\pi^{\frac{1}{2}}\rho^{\frac{3}{2}}}$ [3,12,54].

(A4)
$$\left\{\begin{array}{c} \frac{dC}{dt}=\frac{\alpha }{\chi }\frac{XP}{C^{\frac{3}{2}}}\\ \frac{dX}{dt}=\frac{\eta }{\chi }\frac{XF}{C^{\frac{3}{2}}}\\ \frac{dF}{dt}=\frac{D_{F}K}{\rho \delta^{2}}C\left(F^{*}-\frac{F}{\chi C^{\frac{3}{2}}}\right)-\frac{\eta }{\chi }\frac{XF}{C^{\frac{3}{2}}}\\ \frac{dP}{dt}=\frac{D_{P}K}{\rho \delta^{2}}C\left(P^{*}-\frac{P}{\chi C^{\frac{3}{2}}}\right)-\frac{\alpha }{\chi }\frac{XP}{C^{\frac{3}{2}}} \end{array}\right.$$

If we set *K* = 1 (thus redefining *D_F_* and *D_P_*) and we also appropriately redefine *α* and *η* we obtain:

(A5) $$\left\{\begin{array}{c} \frac{dC}{dt}=\alpha \frac{XP}{C^{\frac{3}{2}}}\\ \frac{dX}{dt}=\eta \frac{XF}{C^{\frac{3}{2}}}\\ \frac{dF}{dt}=\frac{D_{F}}{\rho \delta^{2}}C\left(F^{*}-\frac{F}{\chi C^{\frac{3}{2}}}\right)-\eta \frac{XF}{C^{\frac{3}{2}}}\\ \frac{dP}{dt}=\frac{D_{P}}{\rho \delta^{2}}C\left(P^{*}-\frac{P}{\chi C^{\frac{3}{2}}}\right)-\alpha \frac{XP}{C^{\frac{3}{2}}} \end{array}\right.$$

where *α* is the kinetic coefficient of the reaction that forms the membrane, *η* is the kinetic coefficient of the reaction that forms substance *X* (in this paper these reactions are cleavage and condensation), and *D_F_* and *D_P_* are the diffusion constants across the membrane (see also Table 2 in the main text).
